# Vitamin C Status Correlates with Markers of Metabolic and Cognitive Health in 50-Year-Olds: Findings of the CHALICE Cohort Study

**DOI:** 10.3390/nu9080831

**Published:** 2017-08-03

**Authors:** John F. Pearson, Juliet M. Pullar, Renee Wilson, Janet K. Spittlehouse, Margreet C. M. Vissers, Paula M. L. Skidmore, Jinny Willis, Vicky A. Cameron, Anitra C. Carr

**Affiliations:** 1 Biostatistics and Computational Biology Unit, University of Otago, Christchurch 8140, New Zealand; john.pearson@otago.ac.nz; 2 Department of Pathology, University of Otago, Christchurch 8140, New Zealand; juliet.pullar@otago.ac.nz (J.M.P.); margreet.vissers@otago.ac.nz (M.C.M.V.); 3 Department of Medicine, University of Otago, Christchurch 8140, New Zealand; renee.wilson@postgrad.otago.ac.nz (R.W.); vicky.cameron@otago.ac.nz (V.A.C.); 4 Department of Psychological Medicine, University of Otago, Christchurch 8140, New Zealand; janet.spittlehouse@otago.ac.nz; 5 Department of Human Nutrition, University of Otago, Dunedin 9054, New Zealand; paula.skidmore@otago.ac.nz; 6 Lipid & Diabetes Research Group, Canterbury District Health Board, Christchurch 8140, New Zealand; jinny.willis@cdhb.health.nz

**Keywords:** ascorbate, cognition, HbA1c, insulin, glucose, hypovitaminosis C

## Abstract

A cohort of 50-year-olds from Canterbury, New Zealand (*N* = 404), representative of midlife adults, undertook comprehensive health and dietary assessments. Fasting plasma vitamin C concentrations (*N* = 369) and dietary vitamin C intake (*N* = 250) were determined. The mean plasma vitamin C concentration was 44.2 µmol/L (95% CI 42.4, 46.0); 62% of the cohort had inadequate plasma vitamin C concentrations (i.e., <50 µmol/L), 13% of the cohort had hypovitaminosis C (i.e., <23 µmol/L), and 2.4% had plasma vitamin C concentrations indicating deficiency (i.e., <11 µmol/L). Men had a lower mean plasma vitamin C concentration than women, and a higher percentage of vitamin C inadequacy and deficiency. A higher prevalence of hypovitaminosis C and deficiency was observed in those of lower socio-economic status and in current smokers. Adults with higher vitamin C levels exhibited lower weight, BMI and waist circumference, and better measures of metabolic health, including HbA1c, insulin and triglycerides, all risk factors for type 2 diabetes. Lower levels of mild cognitive impairment were observed in those with the highest plasma vitamin C concentrations. Plasma vitamin C showed a stronger correlation with markers of metabolic health and cognitive impairment than dietary vitamin C.

## 1. Introduction

The role of vitamin C in health and disease has been actively studied since its discovery over 80 years ago [1]. Vitamin C has a number of well-recognized biological functions, all of which depend upon its ability to act as an electron donor [2]. One of the most significant of these is its cofactor activity for a variety of enzymes with critical functions throughout the body. These include the copper-containing monoxygenases dopamine hydroxylase and peptidyl-glycine α-amidating monooxygenase [3] and the Fe (II) and 2-oxoglutarate-dependent family of dioxygenases [4]. The latter is a large and varied family, with a continually expanding membership that includes the collagen prolyl hydroxylases responsible for stabilization of the tertiary structure of collagen, the prolyl and asparaginyl hydroxylases which regulate hypoxia-inducible factors (HIF) activity, and DNA and histone demethylases involved in the epigenetic regulation of gene expression. Vitamin C also functions as a highly effective water-soluble antioxidant, protecting in vivo biomolecules from oxidation [5], and there is good evidence to suggest it is involved in the regeneration of vitamin E in vivo [6,7].

Because humans are unable to synthesize their own vitamin C, it must be obtained from the diet, principally through fruit and vegetable consumption. Inadequate dietary intake results in the potentially fatal deficiency disease, scurvy. As little as 10 mg/day vitamin C is sufficient to prevent overt scurvy [8] and, although scurvy is considered to be relatively rare in Western populations, vitamin C deficiency is the fourth most prevalent nutrient deficiency reported in the United States [9,10]. Hypovitaminosis C (defined as a plasma concentration ≤23 µmol/L) affects a significant proportion of the population, with estimates as large as 15–20% in the United States [9]. Similar data for the New Zealand population are lacking, although dietary vitamin C intake has been used to estimate the prevalence of inadequate intake, defined as not meeting the estimated average requirement (EAR) [11].

The classical symptoms of scurvy, such as joint pain, lassitude, bleeding and ulceration are thought to be due to the loss in activity of the vitamin C-cofactor enzymes, particularly the collagen hydroxylases. It is becoming increasingly acknowledged, however, that vitamin C is required at concentrations above those needed for the prevention of scurvy for the maintenance of good health [12,13]. For example, individuals with hypovitaminosis C are known to present with fatigue, depression and deficiencies in wound healing [14,15], suggesting a requirement for vitamin C status to be above 23 µmol/L in plasma to support these functions. There is also epidemiological evidence to support a role for vitamin C in the prevention of some chronic disease, with intakes >100 mg/day recommended [12]; these intakes will provide adequate plasma levels (i.e., >50 µmol/L) [14,16]. Although the Australasian Recommended Dietary Intake (RDI) for vitamin C is only 45 mg/day, the New Zealand Ministry of Health, in accord with other international bodies, has a suggested dietary target of ~200 mg/day vitamin C for the reduction of chronic disease risk [17]. As the many cofactor functions of vitamin C become more widely understood, epidemiological studies in areas in which its biological activity can be justified are required.

The CHALICE (Canterbury Health, Ageing and Lifecourse) study is a unique New Zealand study comprising a comprehensive database of determinants of health. It has prospectively recruited ~400 fifty-year-olds at random from the electoral roll within the Canterbury region. Participants have undergone extensive health, dietary and social assessments [18]. Here we report on the plasma vitamin C status and dietary vitamin C intake of the participants, and examine the relationships between these measures and a range of health indicators.

## 2. Materials and Methods

### 2.1. Study Population

Participants were from a random sample drawn from the New Zealand electoral roll, recruited to take part in a prospective longitudinal study of health and wellbeing (2010–2013), called the Canterbury Health, Ageing and Lifecourse (CHALICE) study (detailed in [18]). Participants had to be aged 49–51 years, intend to reside within the greater Christchurch area for at least 6 of the next 12 months, live in the community (i.e., not in a prison or a rest home) and be able to complete the assessment (e.g., speak English proficiently). Māori, the indigenous people of New Zealand, were over-sampled so that they represented 15% of the CHALICE study sample. Enrolment statistics estimate that, in 2012, 94.9% of the target population were registered to vote in the Christchurch City Council area [19]. Relative to the rest of New Zealand, the Canterbury area has a slightly higher proportion of people aged ≥40 years and a higher proportion of people living in the least economically deprived national quintile [20]. Ethical approval was obtained from the Upper South A Regional Ethics Committee (URA/10/03/021) and all participants provided written informed consent.

Data were collected during a 4–6 h interview, via self-completed questionnaires and lifestyle diaries, and from blood and urine tests. The full cohort was 404 participants, and the present analysis is based on the 369 participants for whom fasting plasma vitamin C measurements were obtained and a sample of 250 for whom dietary vitamin C intake was determined.

### 2.2. Blood Sample Collection

Fasting blood samples were collected into EDTA anticoagulant tubes and sent to Canterbury Health Laboratories, an International Accreditation New Zealand (IANZ) laboratory, for analysis of biomarkers. Additional fasting samples were centrifuged at 4000 rpm for 10 min at 4 °C to separate plasma, and the plasma stored at −80 °C for vitamin C analysis.

### 2.3. Sample Preparation for Vitamin C Analysis

Stored EDTA-plasma was rapidly thawed and a 500 µL aliquot was treated with an equal volume of ice-cold 0.54 M HPLC-grade perchloric acid solution (containing 100 µmol/L of the metal chelator DTPA) to precipitate protein and stabilize the vitamin C. Samples were mixed, incubated on ice for a few minutes, then centrifuged. A 100 µL aliquot of the deproteinated supernatant was treated with 10 µL of the reducing agent TCEP (100 mg/mL stock) for 2 h at 4 °C to recover any oxidized vitamin C [21]. Samples were further diluted with an equal volume of ice-cold 77 mM perchloric acid/DTPA solution for HPLC analysis.

### 2.4. Vitamin C HPLC Analysis

The total vitamin C content (ascorbic acid plus dehydroascorbic acid) of the samples was determined by HPLC with electrochemical detection as described previously [22]. Samples (20 µL) were separated on a Synergi 4 µ Hydro-RP 80A column 150 mm × 4.6 mm (Phenomenex NZ Ltd, Auckland, New Zealand) using a Dionex Ultimate 3000 HPLC unit (with autosampler chilled to 4 °C and column temperature set at 30 °C) and an ESA coulochem II detector (+200 mV electrode potential and 20 µA sensitivity). The mobile phase comprised 80 mM sodium acetate buffer, pH 4.8, containing DTPA (0.54 mmol/L) and freshly added ion pair reagent n-octylamine (1 µmol/L), delivered at a flow rate of 1.2 mL/min. A standard curve of sodium-L-ascorbate, standardized spectrophotometrically at 245 nm (ε = 9860), was freshly prepared for each HPLC run in 77 mmol/L HPLC-grade perchloric acid containing DTPA (100 µmol/L). Plasma vitamin C content is expressed as µmol/L.

Fasting plasma vitamin C concentrations were classified as follows; deficient <11 μmol/L, marginal 11–23 μmol/L, inadequate 23–50 μmol/L or adequate >50 μmol/L [13,15].

### 2.5. Metabolic and Heart Health Assessments

Metabolic health was assessed by body measurements and fasting blood tests. Participants’ height, weight and waist circumference were taken by the study interviewer, and body mass index (BMI) calculated (kg/m^2^). Fasting blood tests comprised triglycerides, high-density lipoprotein (HDL), glucose, HbA1c and insulin (Canterbury Health Laboratories).

Heart health was assessed by blood pressure and participants had their NZ cardiovascular risk score calculated. Blood pressure measurements were taken while seated. Five year cardiovascular risk (%) was derived according to the New Zealand adaptation of the Framingham risk score; the following variables are included in the calculation: age, gender, systolic blood pressure, diabetic status, smoking history, and total cholesterol to HDL ratio [23].

### 2.6. Dietary Intake Assessment

Participants were asked to complete the Four Day Estimated Food Diary (4DEFD) in the week after their interview; on one weekend day and three weekdays. The 4DEFD included detailed instructions on how to record portion sizes, using common household measures. The completed 4DEFD were checked by a trained nutritionist and additional information obtained from participants where necessary before the data were entered into the nutrient analysis program Kai-culator (version 1.08d, Department of Human Nutrition, University of Otago, Dunedin, New Zealand). Dietary analysis was performed on 250 of the CHALICE participants, who had dietary data entered and cleaned at the time of analysis, for whom the mean daily intake of vitamin C was calculated. Data entry was undertaken by experienced nutritionists and all diaries were further checked for accuracy by one person who also made any necessary changes, to ensure consistency of data entry.

### 2.7. Wellbeing, Depression and Cognition

#### 2.7.1. Mental Wellbeing

The Warwick–Edinburgh Mental Wellbeing Scale (WEMWBS) was used to assess general wellbeing. The 14 item questionnaire aims to measure positive mental health by assessing both aspects of well-being: eudaimonic and hedonic [24].

#### 2.7.2. Depression

During the assessment, trained interviewers used the Mini-International Neuropsychiatric Interview (MINI) for diagnosis of current and past depressive episodes using DSM IV criteria [25].

#### 2.7.3. Cognition

Participants completed the Montreal Cognitive Assessment (MoCA) version 7.1 (original version) [26], a short screening test for mild cognitive impairment. It assesses the cognitive domains of attention and concentration, executive functions, memory, language, visuoconstructional skills, conceptual thinking, calculations, and orientation. A score of 26 or more indicates normal functioning, while a score less than 26 might indicate mild cognitive impairment. MoCA scores were excluded from the analysis if English was the second language or if a previous event (e.g., carbon monoxide poisoning) had affected cognitive ability.

### 2.8. Socio-Economic Status

The Economic Living Standard Index Short Form (ELSI_SF_) was used to assess standard of living [27]. Developed in New Zealand, the ELSI_SF_ assesses a person’s consumption and personal possessions, calculating a total score by combining information from all items of the survey. The ELSI_SF_ scores range from 0–31, with those who score 0–16 described as being in hardship, scores of 17–24 as comfortable and scores of 25 or above as socio-economically good or very good. The ELSI_SF_ has excellent internal consistency (coefficient alpha of 0.88).

### 2.9. Statistical Analyses

Statistical analyses were performed using R 3.3.1 software (R Foundation for Statistical Computing, Vienna, Austria). Univariate tests on continuous variables were t-tests with Satterthwhaite’s adjustment for unequal variances while Wald odds ratios and Fisher exact p-values were calculated for categorical variables. Sample characteristics were compared with census proportions using the chi squared goodness of fit test. All health measures were examined independently for association with vitamin C (plasma vitamin C concentration or dietary vitamin C intake) using linear or logistic regression models. The models fitted the dietary measure, gender (dichotomous), Māori ethnicity (dichotomous) and current smoking (dichotomous). Models were fitted on males and females separately and the whole cohort combined. Modeling assumptions were verified with no material departures observed. For each outcome, the *p* values were adjusted for multiple comparisons using the Benjamini and Yekutieli method. The nominal *p* value for statistical significance is the usual 0.05 or 5% type II error rate. All *p* < 0.1 are shown in the tables with *p* > 0.1 shown as NS (not significant). The odds of currently smoking for those in the lowest socio-economic strata was 3.8 times that of the highest strata (95% CI 1.7–9.0), *p* = 0.002. Similarly the odds of current smoking were 3.4 times higher in the least educated strata than the most educated (95% CI 1.75, 6.54), *p* = 0.0006. To prevent over fitting, socio-economic status and education were not fitted, however smoking acts as a reasonable proxy for population modeling.

## 3. Results

### 3.1. Characteristics of the Study Population

Of the full CHALICE cohort (*N* = 404), 46.8% (189) were male, with 83.7% (338) self-identifying as New Zealand European and 14.9% (60) as Māori (Table 1). The majority of the participants were in the highest ELSI_SF_ category. There were 60 current smokers in the cohort.

Table 1 compares the CHALICE participants to the New Zealand Census 2006 data for similar age and region. The CHALICE participants had higher rates of Māori ethnicity and higher qualifications than the Canterbury average (Table 1), whereas socio-economic status and smoking were within stochastic limits. This suggests the sample is reasonably representative of Canterbury 50-year-olds and hence the national cohort allowing for regional bias.

The CHALICE cohort also had typical levels of health for a community sample (Table 2). Anthropometric measures were close to those of the New Zealand population. Average metabolic and cardiac markers for the cohort were generally within the healthy range. However, the high prevalence of chronic conditions in the New Zealand population was also readily apparent.

### 3.2. Vitamin C Status of the Study Population

**Figure 1:**
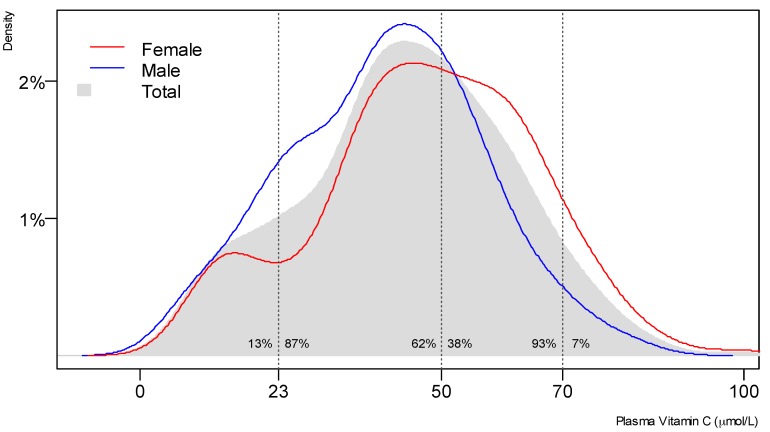
Density plot of plasma vitamin C. Proportion of sample at given vitamin C level; *n* = 369.

Fasting plasma vitamin C measurements were available for 369 of the CHALICE participants. The mean plasma vitamin C concentration was 44.2 μmol/L (95% CI 42.4, 46.0); 62% of the participants were below the adequate level (i.e., 50 μmol/L), and 93% of the participants were below the optimal saturating level (i.e., 70 μmol/L; Figure 1). Ten percent of the cohort had marginal vitamin C concentrations (i.e., 11–23 µmol/L), and vitamin C deficiency, defined as a plasma concentration of <11 µmol/L, was apparent in 2.4% of the cohort (Table 3).

Plasma vitamin C status was substantially lower in men than in women (*p* = 0.005), and it also varied by socio-economic status (*p* = 0.003). For example, 8% of those in the lowest socio-economic category were vitamin C deficient compared to 2.4% of the entire cohort (*n* = 369). Smoking status was also associated with plasma vitamin C status with current smokers having lower vitamin C levels (*p* < 0.001; Table 3).

Study participants with and without vitamin C measurements do not differ significantly by gender, ethnicity, education, socio-economic status, smoking status, waist, weight or BMI (all *p* > 0.13), hence are treated as missing at random.

### 3.3. Associations of Vitamin C Status with Markers of Metabolic and Mental Health

The results of the statistical modeling with plasma vitamin C are summarized in Table 4. Higher plasma vitamin C status was associated with lower weight, BMI and waist circumference in the CHALICE cohort, even after adjustment for gender, ethnicity and current smoking. Of the other markers of metabolic health, plasma vitamin C was negatively associated with blood triglycerides, HbA1c and insulin, and positively associated with HDL levels. However, after multiple adjustment only triglycerides, HbA1c and insulin levels remained significant. No correlation was found between plasma vitamin C and the two indicators of heart health; blood pressure and cardiovascular risk score.

Mild cognitive impairment was assessed by the MoCA test. Higher plasma vitamin C status was correlated with lower mild cognitive impairment, which was maintained after adjustment for gender, ethnicity and current smoking (Table 4). A 1 μmol/L increase in plasma vitamin C was associated with 3% reduced odds of mild cognitive impairment (OR = 0.97, 95% CI = (0.96, 0.99), *p* = 0.004). Indeed, the odds of mild cognitive impairment were twice as high for those below 23 μmol/L plasma vitamin C (OR = 2.1, 95% CI = (1.2, 3.7), *p* = 0.01). Plasma vitamin C status was not associated with wellbeing or depression.

### 3.4. Dietary Vitamin C Intake

Dietary intake analysis was performed on 250 of the CHALICE participants. The average dietary vitamin C intake was 110 mg/day, with 12% falling below the New Zealand recommended dietary intake (RDI, Table 5). There was little effect of gender, ethnicity or socio-economic status on dietary intake. However, those with the lowest educational qualifications tended to have lower dietary vitamin C intake, although this was not quite significant. Current smokers also had a lower dietary intake of vitamin C (*p* < 0.001). Dietary vitamin C intake correlated somewhat less than expected with plasma levels of vitamin C, although the correlation was statistically significant (Pearson’s correlation coefficient *r* = 0.27, *p* = 0.00002).

### 3.5. Associations of Dietary Vitamin C Intake with Markers of Metabolic and Mental Health

There was evidence that higher dietary intake of vitamin C was associated with lower waist circumference and insulin levels, after adjustment for gender, ethnicity and current smoking (Table 6). Glucose and HbA1c levels were inversely associated with dietary vitamin C intake in the initial models, however they did not remain so after correction for multiple comparisons. Higher dietary vitamin C intake was also associated with lower blood pressure, although there was no effect on cardiovascular risk score. There was little association between dietary vitamin C intake and mental health measures, although dietary intake was inversely associated with mild cognitive impairment in the unadjusted model.

## 4. Discussion

These findings were drawn from the first phase of the CHALICE study, a longitudinal observational study of randomly selected 50-year-olds from the Canterbury region, New Zealand in 2010–2013. The comprehensive range of instruments used in the CHALICE study gives a broad picture of the cohort’s health and the agreement between the study data and national demographics provides confidence that the study is representative of the health of 50-year-old New Zealanders in 2010. The cohort has typical levels of metabolic and cardiac markers, with indications of overweight/obesity and hypertension in some individuals. Our study provides new evidence that mid-life adults with higher vitamin C levels exhibited better measures of metabolic health and lower levels of mild cognitive impairment.

In New Zealand, dietary vitamin C intake has been estimated by several comprehensive national dietary surveys, including the 2008/2009 New Zealand Adult Nutrition Survey in which the mean usual adult daily intake was 108 mg based on 24 h dietary recall data [11]. This is close to the average dietary intake of 110 mg/day found in the current study. However, measuring vitamin C concentrations in the body has a number of advantages over dietary intake. It does not rely on participant’s recall of their diet, and takes in all sources of the vitamin, including supplements, and the potential impact of vitamin C losses due to food processing and preparation. More particularly, it accounts for confounders of vitamin C status such as smoking, alcohol consumption, prescription medications and health conditions which may affect turnover of the vitamin [31]. The CHALICE study is the first representative study of plasma vitamin C status within the New Zealand population. Only smaller studies in specific, non-representative groups have measured plasma vitamin C concentrations within the New Zealand population [32,33].

In our study, we found that 2.4% of 50-year-olds were deficient in vitamin C (i.e., <11 µmol/L), putting them at higher risk of developing scurvy and other health effects that may be associated with very low vitamin C status. Men were at greater risk of being deficient than women, and having lower socio-economic status significantly increased risk. Smoking also increased the risk of deficiency, most likely due to increased oxidative stress causing faster turnover of the vitamin [31]. In addition, in our cohort, smokers had a lower dietary intake of vitamin C. Numerous studies have previously shown gender, socio-economic status and smoking to be important predictors of vitamin C status [9,33,34,35,36,37]. A recent study suggests the effect of gender on vitamin C status may be due to the differing fat free mass between men and women, meaning vitamin C is distributed throughout a higher volume in men, leading to lower vitamin C concentrations in the plasma [36].

Data from large international cohorts show similar levels of vitamin C deficiency and hypovitaminosis C to the CHALICE cohort [37,38], although the United States and lower socio-economic groups in the United Kingdom stand out as having higher rates of deficiency [9,34]. In the current study, hypovitaminosis C (i.e., <23 µmol/L) was apparent in 13% of participants, and this increased to 36% for those in the lowest socio-economic category. Symptoms such as decreased mood and energy levels may be observed with hypovitaminosis C, and are possibly related to the role of vitamin C as a cofactor in carnitine and catecholamine neurotransmitter synthesis [3,14]. A high proportion (63%) of our participants had inadequate plasma vitamin C concentrations (i.e., <50 µmol/L). Indeed, very few of our participants, only 7%, had saturating plasma vitamin C status (i.e., >70 µmol/L), implying that current Ministry of Health guidelines recommending consumption of at least five servings of vegetables and fruit per day are ineffective [39]. Since the vitamin C content of fruit and vegetables is quite variable, we suggest that it is important to highlight the consumption of high vitamin C-content fruit and/or vegetables to provide plasma saturation in this age group.

High vitamin C concentrations in the blood were associated with significantly lower weight, waist circumference and BMI, and the effect of plasma vitamin C status was significant enough to survive the correction for multiple comparisons. The association of low vitamin C with obesity in this study replicates results in the literature [35,40,41,42,43,44], and it is apparent that individuals with higher weight require higher intakes of vitamin C to reach adequate vitamin C status [45,46]. We also show that higher plasma vitamin C status is associated with lower circulating levels of blood triglycerides, insulin and HbA1c, associations which survive correction for gender, ethnicity and current smoking. These findings are in agreement with a number of smaller intervention studies that have found inverse relationships of vitamin C with various markers of metabolic health [47,48,49], although others have failed to observe an effect of intervention [50]. Dakhale and coworkers show a small decrease in HbA1c and fasting blood glucose in individuals with type 2 diabetes after vitamin C supplementation of 1 g/day for 12 weeks [51]. Observational studies also provide evidence that low vitamin C status is associated with increased risk of metabolic syndrome [52,53,54].

A role for vitamin C in the prevention or management of diabetes and/or metabolic syndrome has been suggested [47,51,53,54]. Obesity is a major risk factor for diabetes, and it may be that vitamin C has a role in moderating the inflammatory effect of adipose tissue. Vitamin C is thought to have anti-inflammatory activity, decreasing levels of inflammatory markers such as C-reactive protein and pro-inflammatory cytokines, although the exact mechanism(s) responsible for this are unknown [55,56]. Disorders of energy balance and metabolism are common worldwide. For example, in New Zealand, around 241,000 individuals have been diagnosed with diabetes, and significant numbers have undiagnosed diabetes, or pre-diabetes [57]. Further, among people aged over 15 years, 65% of individuals meet the criteria for overweight and obesity [58]. Diet and lifestyle factors are associated with these disorders and represent key modifiable determinants. Interestingly, in the CHALICE cohort there were no consistent significant effects identified between plasma vitamin C status and blood pressure or cardiovascular disease risk, although higher dietary vitamin C intake was associated with decreased blood pressure, an effect that has been observed previously [59].

In this study, we also demonstrate lower levels of mild cognitive impairment in those with high vitamin C status, even after adjustment for gender, ethnicity and smoking. Current smoking was a good proxy for socio-economic status and educational achievement in the model; thus, the relationship with vitamin C status survived correction for these important predictors of cognitive impairment. The odds of mild cognitive impairment were twice as high for those below 23 μmol/L plasma vitamin C concentration. Vitamin C is present at very high concentrations in the brain [60], and animal models have shown that the brain is the last organ to be depleted of the vitamin during prolonged deficiency [61], suggesting an important requirement for vitamin C in the central nervous system. A recent animal study has shown that moderate vitamin C deficiency may play a role in accelerating amyloid plaque accumulation in Alzheimer’s disease, the most common form of dementia [62]. However, epidemiological studies have been inconclusive in regards to whether vitamin C status may affect cognitive decline [63,64] and Alzheimer’s disease specifically [65,66]. Lu and co-workers investigated the relationship between dietary nutrients and mild cognitive impairment in 2892 elderly Chinese participants using the MoCA test, and found that vitamin C intake exhibited a significant protective effect [64]. Our study has the advantage over many in that plasma vitamin C concentrations have been measured; we were not reliant on dietary intake, which may be susceptible to problems with recall ability and the other confounders mentioned above.

In later life, dementia and disorders of cognition are highly prevalent. Even in the CHALICE sample of 50-year-olds, 15% of the sample scored below the recommended cut point on the MoCA. There is considerable interest in the effect of diet on maintaining cognitive function and delaying neuro-degenerative disease in old age. A 2015 study with 37 older healthy adults demonstrated reduced rates of cognitive decline following consumption of orange juice [67]. This was attributed to the high flavanone content of the orange juice, since flavonoids have been associated with reduced rates of cognitive decline [68,69]. However, it is possible that the vitamin C content of the orange juice may have contributed to the observed effect. In support of this premise, studies have shown that supplementation of older adults with the antioxidant vitamins C and E was able to preserve cognitive performance [70,71,72]. Another study, however, found no impact of antioxidant vitamin supplementation on cognition, despite improvements in markers of oxidative stress [73], demonstrating mixed results in the literature. Intervention studies often look for relatively short-term impacts on cognition instruments in response to different nutrient intakes. In contrast, the CHALICE study measured the association of plasma vitamin C status and dietary intake, more likely to be markers of longer-term lifestyle patterns, with a cognitive instrument (MoCA) as an assessment of current mild cognitive impairment.

There are several limitations to our study, notably the observational design, in which associations do not imply causation. Many factors impact on the health status of individuals and groups, including diet, exercise, temperament, behaviors, socio-economic status and genetics. These factors typically interact and correlate with each other, as they do in the CHALICE cohort, with the result that predictors of health outcomes are related (e.g., low blood pressure is associated with low BMI). We have addressed multiple testing issues with the use of corrected *p* values, and multi-collinearity does not affect individual models as each model only has one independent predictor, with the dichotomous covariates having limited capacity to induce collinearity. While we have focused on the associations of vitamin C with health outcomes, these associations could include the effects of unmeasured nutrients associated with vitamin C intake. Dietary vitamin C and plasma vitamin C status did not always correlate with the same health indicators. However, as detailed above, this is likely due to fasting plasma vitamin C concentration being a more accurate indicator of body status.

## 5. Conclusions

The CHALICE cohort of 404 individuals aged 50 years had an average vitamin C intake of ~110 mg/day, which should provide adequate plasma concentrations [14]. Despite this, a significant proportion of the participants had inadequate plasma vitamin C status. This indicates the likely effects of confounding factors, such as chronic disease, on plasma vitamin C status, and suggests that dietary interventions targeting increased consumption of fruit and vegetables, and increased vitamin C intake in particular, are required for this age group. Metabolic health markers were significantly better in participants with higher plasma vitamin C concentrations, even after correction for confounders. The association of high vitamin C concentrations with the reduction in risk of impaired cognition is intriguing and merits further investigation.

## Figures and Tables

**Table 1:** CHALICE participants compared with Census 2006 50–54-year-olds from same region.

		Chalice (*n*, %)		Census 2006 (%)	*p*
Gender	Female	215	53.2	50.9	NS
	Male	189	46.8	49.1	
Ethnicity	Māori	60	14.9	4.5	<0.0001
	NZ European	338	83.7	74.2	
Socio-Economic Status	Low (ELSI_SF_ score 0–16)	30	7.4	8.2	NS
	Medium (ELSI_SF_ score 17–24)	122	30.2	29.4	
	High (ELSI_SF_ score 25–31)	252	62.4	62.5	
Education	No Qualification	53	13.1	23.9	<0.0001
	Secondary School Qualification	110	27.2	35.2	
	Post-secondary	168	41.6	25.6	
	University Degree	73	18.1	15.3	
Current Smoker		60	14.9	16.6	NS

*N* = 404; *p* (χ^2^*_n_*_−1_) > 0.1 shown as not significant, NS.

**Table 2:** Health of CHALICE participants and normal ranges for the New Zealand population.

	Female				Male			
**Body Measurements**	**Mean**	**Min**	**Max**	**NZ Female Mean**	**Mean**	**Min**	**Max**	**NZ Male Mean**
Weight kg	78.6	49.1	149.9	74.8 (73.5–76.1)	88.4	50.8	143.8	88.0 (86.9–89.1)
BMI kg/m^2^	29.1	17.4	63.4	28.1 (27.6–28.6)	28.1	19.2	48.6	28.6 (28.2–28.9)
Waist cm	92.0	63.0	144.0	86.6 (85.5–87.6)	98.3	72.5	148.0	98.4 (97.4–99.3)
**Metabolism**	**Mean**	**Min**	**Max**	**Healthy Range**	**Mean**	**Min**	**Max**	**Healthy Range**
Triglycerides mmol/L	1.3	0.4	11.7	<1.7	1.6	0.4	11.7	<1.7
HDL mmol/L	1.4	0.8	2.7	1.0–2.2	1.2	0.7	1.9	0.9–2.0
Glucose mmol/L	5.1	3.2	10.8	<6.1	5.4	3.7	17.9	<6.1
HbA1c mmol/L	38.2	27.0	74.0	<40	39.9	28.0	102.0	<40
Insulin pmol/L	60.9	10.0	277.0	10–80	61.2	4.0	480.0	10–80
**Heart Health**	**Mean**	**Min**	**Max**	**Healthy Range**	**Mean**	**Min**	**Max**	**Healthy Range**
BP (systolic) mmHg	131.1	104.0	183.7	120	134.2	97.7	185.7	120
BP (diastolic) mmHg	82.5	60.3	106.0	80	85.0	61.0	128.3	80
CVD risk score %	2.5–5	<2.5	20–25	<2.5	5–10	2.5–5	20–25	<2.5
**Mental Health**	**Mean**	**Min**	**Max**		**Mean**	**Min**	**Max**	
Wellbeing	53.0	16	70		52.7	30	70	
Cognition	27.1	19	30		26.6	16	30	
Current Depression *n* (%)	17 (7.9)				12 (6.3)			

BMI: body mass index, HDL: high-density lipoprotein, BP: blood pressure, CVD: Cardiovascular disease. Body measurements compared with New Zealand mean (95% confidence interval) for 45–55 age range [28]. Metabolic and heart health compared with normal healthy range [23,29,30]. Wellbeing measured by Warwick–Edinburgh scale, cognition by MoCA. Current depression is those currently clinically depressed excluding those diagnosed bipolar (*N* = 203 female, 179 male). One female has no waist measurement, three females no fasting metabolic measures, one male no fasting metabolic measures, one male glucose assay failed and two males HbA1c assay failed, otherwise data are for 215 females and 189 males.

**Table 3:** Categories of vitamin C status.

		Plasma Vitamin C		Deficient		Marginal		Inadequate		Adequate		*p*
		Mean	95% CI	*n*	%	*n*	%	*n*	%	*n*	%	
Total		44.2	(42.4, 46.0)	9	2.4	39	10.6	183	49.6	138	37.4	
Gender	Female	47.4	(44.9, 49.9)	2	1.0	20	10.3	85	43.6	88	45.1	0.005
	Male	40.6	(38.2, 43.0)	7	4.0	19	10.9	98	56.3	50	28.7	
Ethnicity	Non Māori	44.5	(42.6, 46.4)	7	2.2	31	9.8	159	50.3	119	37.7	NS
	Māori	42.4	(37.2, 47.6)	2	3.8	8	15.1	24	45.3	19	35.8	
Socio- Economic Status	Low	36.8	(28.3, 45.3)	2	8.0	7	28.0	9	36.0	7	28.0	0.003
	Medium	43.7	(40.3, 47.1)	4	3.5	14	12.3	53	46.5	43	37.7	
	High	45.3	(43.2, 47.4)	3	1.3	18	7.8	121	52.6	88	38.3	
Education	None	38.7	(33.6, 43.9)	3	6.1	6	12.2	26	53.1	14	28.6	NS
	Secondary School	45.9	(42.1, 49.7)	1	1.0	13	12.6	49	47.6	40	38.8	
	Post-secondary	43.1	(40.6, 45.7)	5	3.3	16	10.6	75	49.7	55	36.4	
	University Degree	48.1	(44.4, 51.9)	0	0.0	4	6.1	33	50.0	29	43.9	
Tobacco	Not Current Smoker	45.9	(44.1, 47.8)	6	1.9	26	8.2	157	49.7	127	40.2	<0.001
	Current Smoker	34.1	(29.2, 38.9)	3	5.7	13	24.5	26	49.1	11	20.8	

Plasma vitamin C classified as deficient <11 μmol/L, marginal 11–23 μmol/L, inadequate 23–50 μmol/L or adequate >50 μmol/L; *n* = 369.

**Table 4:** Significant plasma vitamin C effects for body measures, metabolic health and mental health.

	Vitamin C <23 µmol/L (*n* = 47)		Vitamin C >23 µmol/L (*n* = 321)		*p*	*p* Adjusted
	Mean	95% CI	Mean	95% CI		
Body measurements						
Weight	90.3	(83.3, 97.4)	81.7	(79.8, 83.6)	0.024	0.004
BMI	31.4	(28.7, 34.0)	28.1	(27.5, 28.7)	0.021	<0.001
Waist	103.3	(97.6, 108.9)	93.3	(91.8, 94.8)	0.001	<0.001
Metabolism						
Triglycerides	1.8	(1.4, 2.3)	1.4	(1.3, 1.5)	0.061	0.029
HDL	1.3	(1.2, 1.3)	1.4	(1.3, 1.4)	0.033	NS
Glucose	5.6	(5.2, 6.0)	5.2	(5.0, 5.3)	0.072	0.073
HbA1c	42.2	(39.6, 44.8)	38.5	(37.7, 39.3)	0.009	0.015
Insulin	91.0	(68.4, 113.6)	56.3	(51.9, 60.8)	0.004	0.000
Heart health						
BP (systolic)	132.2	(128.0, 136.4)	132.5	(130.8, 134.2)	NS	NS
BP (diastolic)	83.6	(81.0, 86.3)	83.5	(82.4, 84.6)	NS	NS
CVD risk score	5–10%	(<2.5%, 20–25%)	2.5–5%	(3.5–5%, 5–10%)	0.057	NS
Mental Health						
Wellbeing	50.9	(48.4, 53.4)	53.0	(52.0, 53.9)	NS	NS
	*n*	*%*	*n*	*%*		
MCI	17	40.5	66	21.5	0.012	0.02
Current Depression	6	12.5	20	6.2	NS	NS

MCI: Mild Cognitive Impairment indicated by MoCA score <26 for those without excluding conditions. Current depression is for those without Bipolar Disorder. P values less than 0.1 shown otherwise NS: Not Significant. *p* values adjusted for gender, ethnicity and current smoking.

**Table 5:** Categories of dietary vitamin C intake.

		Dietary Vitamin C		Below RDI		RDI-Average		Above Average		*p*
		Mean	95% CI	*n*	%	*n*	%	*n*	%	
Total		109.8	(101.5, 118.1)	30	12	126	50.4	94	37.6	
Gender	Female	107.4	(96.6, 118.2)	13	9.7	73	54.5	48	35.8	NS
	Male	112.6	(99.7, 125.6)	17	14.7	53	45.7	46	39.7	
Ethnicity	Non Māori	112.0	(102.7, 121.2)	22	10.3	111	51.9	81	37.9	NS
	Māori	97.2	(79.6, 114.7)	8	22.2	15	41.7	13	36.1	
Socio-Economic Status	Low	78.8	(54.4, 103.1)	4	26.7	8	53.3	3	20.0	NS
	Medium	105.0	(90.7, 119.2)	12	15.2	36	45.6	31	39.2	
	High	115.3	(104.4, 126.1)	14	9.0	82	52.6	60	38.5	
Education	None	83.5	(64.2, 102.7)	8	28.6	13	46.4	7	25.0	0.1
	Secondary School	117.1	(98.4, 135.7)	6	10.0	32	53.3	22	36.7	
	Post-secondary	108.6	(97.1, 120.1)	11	9.9	59	53.2	41	36.9	
	University Degree	118.4	(97.6, 139.2)	5	9.8	22	43.1	24	47.1	
Tobacco	Not Current Smoker	114.1	(105.3, 122.8)	20	9.0	112	50.7	89	40.3	<0.001
	Current Smoker	77.5	(54.6, 100.5)	10	34.5	14	48.3	5	17.2	

The cut-off values for the vitamin C categories are as follows: New Zealand recommended dietary intake is 45 mg/day, the average New Zealand intake is 109 mg/day for men and 106 mg/day for women [11]; *n* = 250.

**Table 6:** Significant dietary vitamin C effects based on average intake for body measures, metabolic health and heart health.

	Intake < Average (*n* = 147)		Intake > Average (*n* = 103)		*p*	*p* Adjusted
	Mean	95% CI	Mean	95% CI		
Body measurements						
Weight	82.2	(79.2, 85.3)	79.8	(76.4, 83.3)	NS	NS
BMI	28.5	(27.4, 29.6)	27.2	(26.2, 28.1)	0.08	0.063
Waist	94.6	(92.2, 97.0)	91.2	(88.5, 93.8)	0.06	0.047
Metabolism						
Triglycerides	1.4	(1.3, 1.5)	1.3	(1.1, 1.6)	NS	NS
HDL	1.4	(1.3, 1.4)	1.4	(1.3, 1.4)	NS	NS
Glucose	5.3	(5.1, 5.5)	5.0	(4.9, 5.2)	0.03	0.078
HbA1c	39.6	(38.3, 41.0)	37.8	(36.9, 38.7)	0.03	NS
Insulin	64.6	(55.5, 73.6)	52.3	(44.3, 60.3)	0.05	0.041
Heart health						
BP (systolic)	135.0	(132.5, 137.5)	130.6	(127.4, 133.8)	0.03	0.016
BP (diastolic)	85.2	(83.6, 86.7)	82.3	(80.4, 84.1)	0.02	0.007
CVD risk score	2.8	(2.6, 3.0)	2.6	(2.2, 2.9)	NS	NS
Mental Health						
Wellbeing	52.5	(51.1, 53.8)	52.9	(51.3, 54.4)	NS	NS
	***n***	***%***	***n***	***%***		
MCI	36	24.5	14	13.6	0.04	NS
Current Depression	13	8.8	4	3.9	NS	NS

MCI Mild Cognitive Impairment indicated by MoCA score <26 for those without excluding conditions. Current depression is for those without Bipolar Disorder. *p* values less than 0.1 shown otherwise NS: Not Significant. *p* values adjusted for gender, ethnicity and current smoking. Average is New Zealand average of 109 mg/day for men, 106 mg/day for women [11].
